# Development and validation of a risk score for predicting clinical success after endobiliary stenting for malignant biliary obstruction

**DOI:** 10.1371/journal.pone.0272918

**Published:** 2022-08-19

**Authors:** Nonthalee Pausawasdi, Panotpol Termsinsuk, Phunchai Charatcharoenwitthaya, Julajak Limsrivilai, Uayporn Kaosombatwattana

**Affiliations:** 1 Siriraj GI Endoscopy Center, Faculty of Medicine Siriraj Hospital, Mahidol University, Bangkok, Thailand; 2 Division of Gastroenterology, Department of Medicine, Faculty of Medicine Siriraj Hospital, Mahidol University, Bangkok, Thailand; Humanitas Clinical and Research Center - IRRCS, ITALY

## Abstract

**Background:**

Endoscopic drainage is the primary treatment for unresectable malignant biliary obstruction (MBO). This study developed and validated a pre-endoscopic predictive score for clinical success after stent placement.

**Methods:**

Patients with unresectable MBO undergoing ERCP-guided endobiliary stent placement between 2007 and 2017 were randomly divided into derivation (n = 383) and validation (n = 128) cohorts. To develop the risk score, clinical parameters were built by logistic regression to predict (1) ≥ 50% total bilirubin (TB) resolution within 2 weeks and (2) bilirubin normalization (TB level <1.2 mg/dL) within 6 weeks following stenting. The scoring scheme was applied to the validation cohort to test its performance.

**Results:**

A ≥ 50% TB resolution within 2 weeks was shown in 70.5% of cases. The risk scoring scheme had areas under the receiver operating characteristic curve (AUROC) of 0.70 (95% CI, 0.64–0.76) and 0.67 (95% CI, 0.57–0.77) in the derivation and validation cohorts, respectively. Thirty-one percent had TB normalization within 6 weeks after stenting. Significant predictors for TB normalization were extrahepatic biliary obstruction (odds ratio [OR] = 2.35), pre-endoscopic TB level (OR = 0.88), and stent type (OR = 0.42). The AUROC of a risk score for predicting TB normalization within 6 weeks was 0.78 (95% CI, 0.72–0.83) and 0.76 (95% CI, 0.67–0.86) in the derivation and validation cohorts, respectively. A score > 1.30 yielded a specificity of 98% and a positive predictive value of 84% for predicting TB normalization. A score of < -4.18 provided a sensitivity of 80%–90% and a negative predictive value of 90%–93% for predicting the absence of TB normalization.

**Conclusions:**

The pre-endoscopic scoring system comprising biliary obstruction level, liver biochemistry, and type of stent provides prediction indices for TB normalization within 6 weeks after stenting. This scheme may help endoscopists identify patients with unresectable MBO suited for palliative stenting.

## Introduction

The common causes of malignant biliary obstruction (MBO) include cholangiocarcinoma, pancreatic cancer, gallbladder cancer, lymphoma, and metastatic lymph nodes [1]. The prognosis is grave in unresectable diseases, such as advanced cholangiocarcinoma, with a 5-year survival rate of less than 5% [2, 3]. Quality of life is generally poor due to unpleasant symptoms such as pruritus, malaise, anorexia, weight loss, and jaundice [4–7]. Hyperbilirubinemia of more than 2 mg/dL delays the initiation of chemotherapy due to an increased risk of drug toxicity [8–10].

Palliative biliary decompression with endoscopic retrograde cholangiopancreatography (ERCP)-guided endobiliary stent placement is the primary treatment for unresectable MBO. This technique provides 90%–95% technical success, lower adverse event rates, fewer hospitalizations, lower total costs, and better survival than percutaneous transhepatic biliary drainage (PTBD) [11–16]. In addition to technical success, clinical success is also an important outcome after endobiliary stent placement. MBO patients who achieve clinical success have a longer survival time and are more eligible for chemotherapy than those without clinical success after stenting [17, 18]. However, the definition of clinical success is not well established. Weston et al. defined “clinical success” as a regression of a post-stenting total bilirubin (TB) level of less than 2 mg/dL within 3 weeks if the pre-stenting TB level was lower than 10 mg/dL or within 6 weeks if the pre-stenting TB level was higher than 10 mg/dL [18]. The clinical success in a randomized trial of patients with hilar cholangiocarcinoma (INTERCPT trial) was defined by a 50% reduction in post-stenting TB levels from baseline within 3 weeks after endobiliary stent placement [19].

The placement of an endobiliary stent generally improves the quality of life and enables chemotherapy in unresectable MBO patients [20–22]. Nevertheless, more than 10% of patients do not respond to endobiliary stents [23]. Studies have been conducted to explore factors influencing clinical response after stenting. At least 50% of the liver volume must be drained in hilar cholangiocarcinoma to obtain a 50% reduction in serum bilirubin 30 days after endobiliary stent placement [24]. However, the predictors of clinical failure after endobiliary stent placement are a high pre-stent bilirubin level, the presence of cholangitis, diffuse liver metastasis, a prolonged international normalized ratio, and malignant ascites [25–30].

To optimize palliative drainage strategies, it is essential to identify the parameters associated with the regression of serum bilirubin levels after endobiliary stent placement. Therefore, the present study aimed to develop and validate a pre-endoscopic score for identifying clinical success after endobiliary stent placement in patients with unresectable MBO.

## Materials and methods

### Ethics statement

The Institutional Review Board of the Faculty of Medicine Siriraj Hospital approved the study protocol, which met the ethical guidelines of the 1975 Helsinki Declaration (approval number 097/2019). Informed written consent was waived, given the retrospective nature of the study.

### Study population

A retrospective review of the ERCP database of a large tertiary care center was conducted. Eligible subjects were patients who underwent their first ERCP with endobiliary stent placement for unresectable MBO between January 2007 and December 2017. The criteria for unresectable MBO were as follows:

- Inoperable, locally advanced disease due to major vascular involvement (specifically, bilateral or contralateral portal vein, hepatic artery, or secondary biliary radicle involvement)
- Bismuth–Corlette type IV hilar cholangiocarcinoma
- Distant organ or lymph node metastasis
- Unfit for surgery due to significant comorbid conditions and poor functional status.

A total of 1334 patients with unresectable MBO were identified. Of these patients, 823 were excluded. The reasons were incomplete data (n = 417), concomitant other biliary drainage procedures (n = 128), previous endobiliary stent placement (n = 73), failed cannulation or no stent placement (n = 64), advanced cirrhosis (n = 62), death within 2 weeks after endobiliary stent placement (n = 30), absence of jaundice (n = 25), receiving chemotherapy prior to stenting (n = 18), and percutaneous cholecystostomy (n = 6). The remaining 511 patients were enrolled and randomly divided in a 3:1 fashion into a derivation cohort (n = 383) and a validation cohort (n = 128) using computer generation.

All relevant clinical and laboratory data before and after ERCP were collected. In addition, cholangiographic findings and cross-sectional images, including abdominal multi-detector computer tomography and magnetic resonance imaging were reviewed. The diagnosis of malignant obstruction was based on either histopathology alone or combined imaging and clinical follow-up in cases where histology could not be obtained.

### Definition of clinical success and outcome measurements

The primary outcome of our study was clinical success after endobiliary stent placement. “clinical success” was signified by the following:

- a decrease in TB levels of more than 50% within 2 weeks after stenting
- TB normalization, defined by regression of TB levels below 1.2 mg/dL within 6 weeks after stenting

The selection of the TB reduction cutoff of ≥ 50% was based on the definition of clinical success in the INTERCPT trial [19]. The cutoff of 1.2 mg/dL is lower than the acceptable TB level for proceeding with chemotherapy. However, we selected it because a normalized TB level usually contributes to the resolution of unpleasant symptoms resulting from hyperbilirubinemia (such as pruritus, malaise, anorexia, and weight loss). These resolutions eventually improve the quality of life of unresectable MBO patients. The duration of 6 weeks was based on the work of Weston and colleagues [18].

### Statistical analysis

Categorical variables are expressed as frequencies and percentages. Continuous variables are expressed as the mean ± standard deviation for normally distributed continuous data and the median and interquartile range for skewed distribution data. Groups were compared using the Chi^2^ test or Fisher’s exact test for categorical variables and the independent *t*-test or Mann–Whitney U test for continuous variables. Differences were considered statistically significant when probability (*P*) values < 0.05.

The clinical variables that influenced the study outcomes of patients with and without clinical success were identified and compared using univariate logistic regression. The variables were summarized with odds ratios (ORs) and 95% confidence intervals (CIs). The significant variables from this step were selected for a multivariate logistic regression model and presented as Model 1. Because of the long recruitment period of the current study, the calendar year of endobiliary stent placement was considered a variable in the logistic regression models to control the potential effect on the outcomes and demonstrated in Model 2. The coefficients estimated for each factor, which provided the best predictive ability, were relative weights to compute the pre-endoscopic risk score. The calibration of the pre-endoscopic risk score was assessed by comparing the actual observed risk and the average probability of clinical success predicted by the score. The Hosmer–Lemeshow test was used to assess the corresponding goodness-of-fit.

The discriminative power of the developed prediction score was estimated by calculating the area under a receiver operating characteristic curve (AUROC). Cutoffs were selected to categorize patients into 3 groups: “low,” “intermediate,” and “high likelihood” of clinical success after endobiliary stent placement. In addition, sensitivity, specificity, positive predictive value (PPV), negative predictive value (NPV), positive likelihood ratio (LR+), and negative likelihood ratio (LR−) were determined. All statistical analyses were performed using PASW Statistics for Windows, version 18 (SPSS Inc., Chicago, IL, USA).

## Results

### Baseline characteristics

Of the 511 unresectable MBO patients, 383 and 128 were randomly assigned to the derivation and validation cohorts, respectively. The mean age of the derivation cohort was 63.4 years, and 196 patients (51.1%) were men. Cholangiocarcinoma was the most common diagnosis, accounting for 48.3% of cases, followed by pancreatic cancer (30.8%). The most common clinical presentation was jaundice (89%), followed by abdominal pain (55.9%). The median pre-endoscopic TB level was 18.1 mg/dL, and the mean pre-endoscopic serum albumin level was 3.2 g/dL. Most patients had extrahepatic obstruction (58.5%) and hilar obstruction (37.3%). Evidence of distant metastasis was observed in 57% of the cases. Thirty-four percent of the patients had liver metastasis, and 12.8% had peritoneal carcinomatosis.

Most of the patients who underwent endoscopic biliary decompression had only 1 stent placed (94.5%), and the metallic stent was more commonly used than the plastic stent (60.3% vs 34.2%). Seventy percent of cases had more than a 50% reduction in TB within 2 weeks, whereas 31.2% of cases achieved TB normalization within 6 weeks after stenting. Table 1 compares the derivation and validation cohorts’ baseline characteristics, imaging findings, and endoscopic interventions.

**Table 1:** Comparison of baseline characteristics between patients in the derivation and validation cohorts.

Characteristics	Derivation cohort (N = 383)	Validation cohort (N = 128)	*P* value
Male gender, n (%)	196 (51.2%)	66 (51.6%)	0.939
Age, years	63.4 ± 13.1	64.5 ± 12.2	0.812
**Type of malignancy**			
Cholangiocarcinoma, n (%)	185 (48.3%)	56 (43.8%)	0.372
Intrahepatic cholangiocarcinoma	31 (16.8%)	12 (21.4%)	0.651
Hilar cholangiocarcinoma	108 (58.4%)	30 (53.6%)	0.769
Extrahepatic cholangiocarcinoma	46 (24.9%)	14 (25.0%)	0.744
Pancreatic cancer	118 (30.8%)	42 (32.8%)	0.672
Gallbladder cancer	32 (8.4%)	13 (10.2%)	0.534
Ampullary cancer	14 (3.7%)	5 (3.9%)	0.996
Clinical presentation			
Abdominal pain	214 (55.9%)	71 (55.5%)	0.936
Jaundice	341 (89.0%)	115 (89.8%)	0.798
Fever	23 (6.0%)	14 (10.9%)	0.062
Ascending cholangitis	75 (19.6%)	21 (16.4%)	0.426
**Pre-endoscopic laboratory**			
Hemoglobin, g/dL	10.6 ± 2.9	10.6 ± 1.7	0.558
Platelet x 10^3^/microliter	319 (258–404)	326 (268–415)	0.527
INR	1.4 ± 0.5	1.2 ± 1.1	0.373
Total bilirubin, mg/dl	18.1 (12.0–25.7)	20.1 (13.1–26.0)	0.160
Albumin, g/dL	3.2 ± 0.6	3.2 ± 0.6	0.379
Alkaline phosphatase, IU/L	473 (284–702)	443 (298–663)	0.701
Creatinine, mg/dL	0.8 (0.6–0.9)	0.9 (0.7–0.8)	0.889
**Cross-sectional imaging**			
Size of obstructive tumor, cm	3.6 (2.5–5.4)	4.0 (2.8–5.4)	0.329
Hilar obstruction, n (%)	143 (37.3%)	50 (39.1%)	0.727
Non-hilar obstruction, n (%)	240 (62.7%)	78 (60.9%)	0.089
Intrahepatic obstruction	15 (3.9%)	7 (5.5%)	0.454
Extrahepatic obstruction	224 (58.5%)	71 (55.5%)	0.550
Combined obstruction	15 (3.9%)	5 (3.9%)	0.996
Portal vein invasion, n (%)	112 (29.2%)	35 (27.3%)	0.681
Distant metastasis, n (%)	220 (57.4%)	72 (56.3%)	0.814
Liver metastasis, n (%)	130 (33.9%)	46 (35.9%)	0.681
Peritoneal carcinomatosis, n (%)	49 (12.8%)	8 (6.3%)	**0.042**
Endoscopic intervention, n (%)			
One stent placement	362 (94.5%)	117 (91.4%)	0.209
Plastic stent placement	131 (34.2%)	42 (32.8%)	0.955
Metallic stent placement	231 (60.3%)	75 (58.6%)	0.955
Uncovered SEMS	219 (57.2%)	74 (57.8%)	0.596
Fully covered SEMS	8 (2.1%)	1 (1.6%)	0.695
Partially covered SEMS	4 (1.0%)	0 (0)	0.576
Two-stent placements	21 (5.5%)	11 (8.6%)	0.209
Two metallic stents	13 (3.4%)	6 (4.7%)	0.721
Two plastic stents	7 (1.8%)	3 (2.3%)	1.000
One metallic and one plastic stent	1 (0.3%)	2 (1.6%)	0.266
Post-stenting outcomes			
≥ 50% reduction of TB within 2 weeks	270 (70.5%)	95 (74.2%)	0.420
TB normalization within 6 weeks	91 (31.2%)	25 (26.9%)	0.345
Chemotherapy after stenting, n (%)	65 (17%)	15 (11.7%)	0.157

INR, international normalized ratio; SEMS, self-expandable metallic stent; TB, total bilirubin

Data are presented as the mean ± standard deviation, median (interquartile range), or number (proportion) of patients with a condition.

## Reduction of total bilirubin within 2 weeks after stent placement

### Identifying the predictive factors

In the derivation cohort, 270 patients (70.4%) saw a more than 50% improvement in their serum TB levels within 2 weeks after endobiliary stent placement. Patients with extrahepatic bile duct obstruction due to pancreatic cancer and ampullary cancer were more likely to achieve jaundice regression after stenting than hilar cholangiocarcinoma. The presence of portal vein invasion and peritoneal carcinomatosis on imaging was significantly associated with no resolution of hyperbilirubinemia after placement of endobiliary stents (S1 Table). Additionally, uncovered self-expandable metallic stents were used more frequently in patients who achieved a 50% reduction in TB within 2 weeks than in those without (61.9% vs 46.0%; *P* = 0.006). In contrast, plastic stents were used more often in those without TB improvement (46.0% vs 29.3%; *P* = 0.001). The duration of stent patency was significantly longer in the patients with a reduction in TB level (83.0 vs 28.5 days; *P*<0.001). This finding suggests that the metallic stents had better patency than the plastic stents (S2 Table).

Table 2 summarizes the univariate and multivariate analyses of clinical variables for predicting a TB level reduction of more than 50% within 2 weeks after endobiliary stent placement. In the multivariate regression model, the significant baseline factors were extrahepatic obstruction (OR, 2.60; 95% CI, 1.63–4.13), pre-endoscopic alkaline phosphatase (ALP) level (OR, 1.09; 95% CI, 1.01–1.18), peritoneal carcinomatosis (OR, 0.47; 95% CI, 0.25–0.91), and the presence of a plastic stent (OR, 0.43; 95% CI, 0.27–0.70). These factors are shown in Model 1. These variables remained significant after an adjustment was made for the calendar year of endobiliary intervention to recognize the long recruitment period (Model 2).

**Table 2:** Univariate and multivariate analyses of baseline variables for predicting 50% total bilirubin reduction within 2 weeks after stenting in the derivation cohort.

**Univariate analysis**				
**Characteristics**	**Regression coefficient**	**Standard error**	**Unadjusted OR (95% CI)**	***P* value**
Pancreatic cancer	0.753	0.266	2.12 (1.26–3.58)	**0.005**
Pre-endoscopic ALP (times above ULN)	0.081	0.039	1.09 (1.00–1.17)	**0.039**
Extrahepatic biliary obstruction	1.000	0.230	2.72 (1.73–4.27)	**< 0.001**
Hilar obstruction	-0.883	0.230	0.41 (0.26–0.65)	**< 0.001**
Cholangiocarcinoma	-0.629	0.227	0.53 (0.34–0.83)	**0.006**
Endobiliary drainage with plastic stent	-0.759	0.230	0.47 (0.30–0.73)	**0.001**
Peritoneal carcinomatosis	-0.679	0.314	0.51 (0.27–0.94)	**0.030**
Combined intra and extrahepatic obstruction	-1.337	0.540	0.26 (0.09–0.76)	**0.013**
**Multivariate analysis**				
**Characteristics**	**Regression coefficient**	**Standard error**	**Adjusted OR (95% CI)**	***P* value**
**Model 1** ^a^				
Extrahepatic biliary obstruction	0.954	0.237	2.60 (1.63–4.13)	**< 0.001**
Pre-endoscopic ALP (times above ULN)	0.088	0.041	1.09 (1.01–1.18)	**0.032**
Peritoneal carcinomatosis	-0.749	0.336	0.47 (0.25–0.91)	**0.026**
Endobiliary drainage with plastic stent	-0.834	0.243	0.43 (0.27–0.70)	**0.001**
**Model 2** ^b^				
Extrahepatic biliary obstruction	0.951	0.237	2.59 (1.63–4.12)	**< 0.001**
Pre-endoscopic ALP (times above ULN)	0.088	0.041	1.09 (1.01–1.18)	**0.033**
Peritoneal carcinomatosis	-0.747	0.336	0.47 (0.25–0.92)	**0.026**
Endobiliary drainage with plastic stent	-0.807	0.250	0.45 (0.27–0.73)	**0.001**

95% CI, 95% confidence interval; ALP, alkaline phosphatase; OR, odds ratio; ULN, upper limit of normal.

^a^ Model 1 includes the baseline factors that were significant in univariate analysis.

^b^ Model 2 includes the factors from Model 1 plus the calendar year of the endobiliary intervention.

### Development and validation of the predictive score

The risk scoring system was developed using the baseline factors significantly associated with a ≥ 50% decrease in TB after endobiliary stent placement. Improved serum TB levels within 2 weeks post-stent placement were associated with extrahepatic biliary obstruction, pre-endoscopic ALP level, peritoneal carcinomatosis, and the type of endobiliary stent, with coefficients of 0.954, 0.088, -0.749, and -0.834, respectively. The equation was as follows:

Bilirubin improvement scoring system = 0.38 + (0.954 x extrahepatic biliary obstruction [yes = 1, no = 0]) + (0.088 x pre-endoscopic ALP level [times of ULN]) + (-0.749 x peritoneal carcinomatosis [yes = 1, no = 0]) + (-0.834 x the type of endobiliary stent [plastic stent = 1, metallic stent = 0])

with “1” and “0” used for the presence and absence of each factor.

The coefficients in the equation were multiplied by five to simplify the score. Thus, the simplified risk score for predicting early clinical success could be calculated from this:

2 + (5 x extrahepatic biliary obstruction [yes = 1, no = 0]) + (0.5 x pre-endoscopic ALP level [times of ULN]) + (-4 x peritoneal carcinomatosis [yes = 1, no = 0]) + (-4 x the type of endobiliary stent [plastic stent = 1, metallic stent = 0])

An online score calculator is available at https://siendoscopy.wixsite.com/tbimprovement.

**Fig 1:**
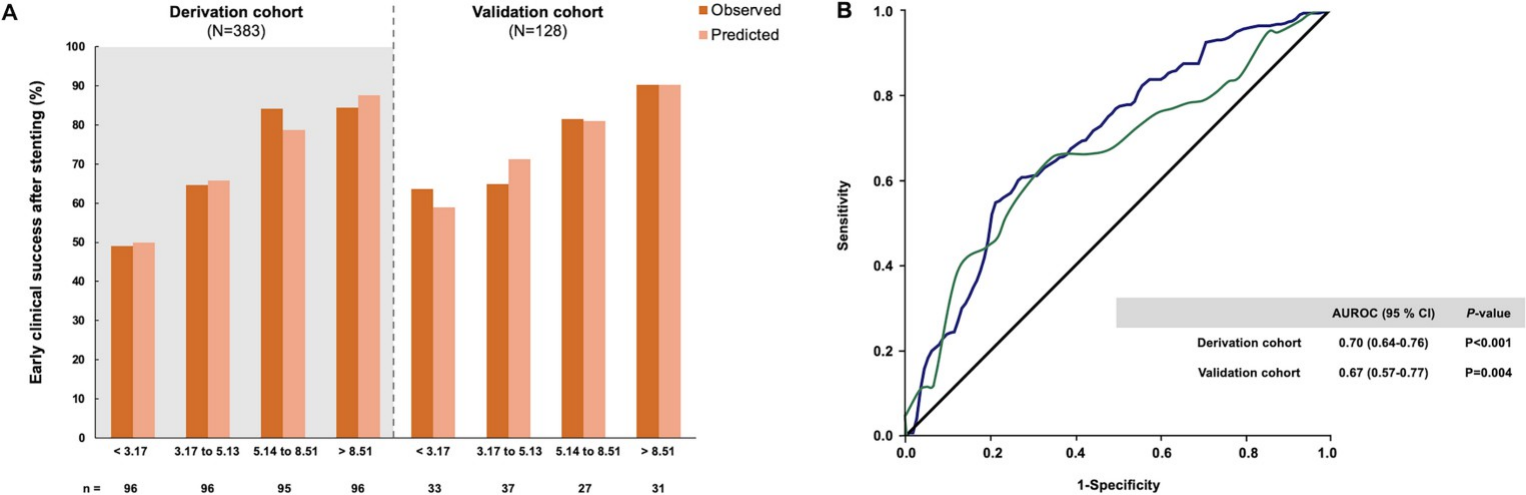
The probability of ≥ 50% total bilirubin reduction within 2 weeks after endoscopic drainage and diagnostic accuracy of the risk score in the derivation and validation cohorts. (A) The observed and predicted clinical success rates are based on the approximate quartiles of the risk score. (B) The area under the receiver operating characteristic curve of the risk score.

The observed and predicted probability of a 50% reduction in TB levels within 2 weeks after stenting are illustrated in Fig 1A according to the approximate quartiles of the clinical risk score. The predicted and observed probabilities were similar across the quartiles of the clinical risk score in the derivation cohort (Hosmer–Lemeshow χ^2^ = 9.58; *P* = 0.30) and the validation cohort (Hosmer–Lemeshow χ^2^ = 7.72; *P* = 0.46).

The scores in the derivation cohort ranged from -6.09 to 17.9. The AUROC was 0.70 (95% CI, 0.64–0.76). The validation analysis included 128 patients with unresectable MBO, and the AUROC of the score predicting early clinical success in the validation cohort was 0.67 (95% CI, 0.57–0.77; Fig 1B). The optimal cutoff of 4.07 had a sensitivity of 67%–70%, specificity of 52%–57%, positive likelihood ratio (+LR) of 1.39–1.61, and negative likelihood ratio (-LR) of 0.53–0.63 for predicting an improvement of the TB level within 2 weeks after endobiliary stent placement (S3 Table).

## Bilirubin normalization within 6 weeks after stent placement

### Identifying the predictive factors

Eighty-two patients died, and 44 were lost to follow-up within 2 to 6 weeks after endoscopic drainage. Thus, 385 patients were included in the analysis. The derivation cohort had 292 patients, while the validation cohort had 93 patients.

Ninety-one patients (31.2%) in the derivation cohort achieved TB normalization (defined by a reduction in TB levels below 1.2 mg/dL within 6 weeks after endobiliary stent placement). The 50% reduction in TB levels within 2 weeks was significantly associated with TB normalization within 6 weeks. However, 66.2% of patients who had early improvement in TB levels within 2 weeks failed to achieve TB normalization by 6 weeks, and 59 patients (15.4%) died between 2 and 6 weeks after stenting. Patients with cholangiocarcinoma were less likely to achieve TB normalization than those with other types of malignancy. Extrahepatic obstruction was significantly associated with TB normalization, whereas hilar obstruction was associated with no TB normalization. Most patients presented with jaundice (84.6%) and abdominal pain (50.5%). Low pre-endoscopic TB levels and high albumin levels were significantly associated with TB normalization after stenting. Patients who achieved TB normalization after stenting were more eligible for chemotherapy than those without (39.6% vs 12.4%; *P*<0.001; S4 Table).

Metallic stents, especially uncovered SEMSs, for palliative biliary decompression were significantly associated with TB normalization. In contrast, most patients with plastic stents failed to achieve TB normalization. Moreover, the metallic stent was more effective than the plastic stent for biliary drainage, as demonstrated by the lower stent dysfunction rate and longer stent patency time (S5 Table).

In the univariate analysis (Table 3), the significant variables for experiencing TB normalization after endobiliary stent placement were pancreatic cancer (*P* = 0.031), high pre-endoscopic serum albumin levels (*P* = 0.009), extrahepatic obstruction (*P* = 0.026), and the type of endobiliary stent (*P* = 0.022). However, the following were inversely associated with TB normalization: jaundice at presentation (*P* = 0.040), pre-endoscopic TB levels (*P*<0.001), low pre-endoscopic serum albumin levels (*P* = 0.009), low pre-endoscopic international normalized ratio (*P* = 0.014), and hilar obstruction on cross-sectional imaging (*P* = 0.036).

**Table 3:** Univariate and multivariate analysis of baseline variables for predicting bilirubin normalization within 6 weeks after stenting in the derivation cohort.

**Univariate analysis**				
**Characteristics**	**Regression coefficient**	**Standard error**	**Unadjusted OR (95% CI)**	***P* value**
Pancreatic cancer	0.573	0.265	1.77 (1.06–2.98)	**0.031**
Extrahepatic biliary obstruction	0.590	0.266	1.80 (1.07–3.04)	**0.026**
Hilar obstruction on cross-sectional imaging	-0.561	0.268	0.57 (0.34–0.97)	**0.036**
Pre-endoscopic serum albumin level	0.575	0.221	1.78 (1.15–2.74)	**0.009**
Pre-endoscopic total bilirubin level	-0.116	0.018	0.89 (0.86–0.92)	**< 0.001**
Pre-endoscopic INR level	-0.973	0.394	0.38 (0.18–0.82)	**0.014**
Endobiliary drainage with plastic stent	-0.626	0.272	0.54 (0.31–0.91)	**0.022**
Jaundice at presentation	-0.813	0.396	0.44 (0.20–0.96)	**0.040**
**Multivariate analysis**				
**Characteristics**	**Regression coefficient**	**Standard error**	**Adjusted OR (95% CI)**	***P* value**
**Model 1** ^a^				
Extrahepatic biliary obstruction	0.852	0.307	2.35 (1.29–4.28)	**0.006**
Pre-endoscopic total bilirubin level	-0.129	0.020	0.88 (0.85–0.91)	**< 0.001**
Endobiliary drainage with plastic stent	-0.874	0.317	0.42 (0.22–0.78)	**0.006**
**Model 2** ^b^				
Extrahepatic biliary obstruction	0.878	0.287	2.41 (1.37–4.23)	**0.002**
Pre-endoscopic total bilirubin level	-0.124	0.510	0.88 (0.85–0.92)	**< 0.001**
Endobiliary drainage with plastic stent	-0.609	0.309	0.54 (0.30–1.00)	**0.049**

95% CI, 95% confidence interval; OR, odds ratio

^a^ Model 1 includes the baseline factors that were significant in univariate analysis.

^b^ Model 2 includes the factors from Model 1 plus the calendar year of the endobiliary intervention.

In the multivariate regression model (Table 3), the independent predictors of TB normalization were extrahepatic obstruction (OR 2.35; 95% CI, 1.29–4.28), pre-endoscopic TB levels (OR 0.88; 95% CI, 0.85–0.91), and endobiliary drainage with plastic stent (OR 0.42; 95% CI, 0.22–0.78). These are shown in Model 1. These parameters remained significant when the calendar year of endobiliary intervention was factored into Model 2.

## Development and validation of the predictive score

Extrahepatic biliary obstruction, pre-endoscopic TB levels, and the type of endobiliary stent were independent variables for predicting TB normalization within 6 weeks after endobiliary stent placement. Their coefficients were 0.852, -0.129, and -0.874, respectively. The equation for predicting TB normalization was as follows:

1.086 + (-0.129 x pre-endoscopic serum TB levels) + (0.852 x extrahepatic biliary obstruction [yes = 1, no = 0]) + (-0.874 x the type of endobiliary stent [plastic stent = 1, metallic stent = 0])

with “1” and “0” used to denote the presence and absence of each factor.

The coefficients in the equation were multiplied by two to simplify the score. Thus, the simplified scoring system for the prediction of TB normalization was calculated from this:

2 + (-0.3 x pre-endoscopic serum TB levels) + (2 x extrahepatic biliary obstruction [yes = 1, no = 0]) + (-2 x the type of endobiliary stent [plastic stent = 1, metallic stent = 0])

An online score calculator is available at https://siendoscopy.wixsite.com/tbimprovement.

**Fig 2:**
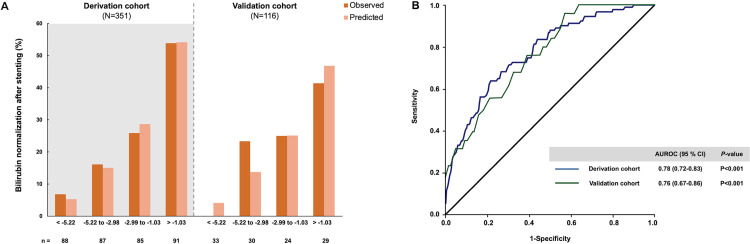
The probability of bilirubin normalization and diagnostic accuracy of the risk score in the derivation and validation cohorts. (A) The observed and predicted rates of bilirubin normalization after endobiliary stent placement according to the approximate quartiles of the risk score. (B) The area under the receiver operating characteristic curve of the risk score for predicting bilirubin normalization.

The observed and predicted probability of TB normalization within 6 weeks post-endobiliary stenting are depicted in Fig 2A according to the approximate quartiles of the clinical risk score. The predicted and observed probabilities of TB normalization were similar across the quartiles of the clinical risk score in the derivation cohort (Hosmer–Lemeshow χ^2^ = 6.86; *P* = 0.55) and the validation cohort (Hosmer–Lemeshow χ^2^ = 6.69; *P* = 0.57). These results signified good performance of the TB normalization score throughout the whole score-value range.

The score in the derivation cohort ranged from -13.6 to 3.52, with an AUROC of 0.78 (95% CI, 0.72–0.83). The validation analysis included 116 patients with unresectable MBO. The AUROC of a score for predicting TB normalization in the validation cohort was 0.76 (95% CI, 0.67–0.86), similar to that of the derivation cohort and demonstrating the risk score’s reliability (Fig 2B). The optimal cutoff of -2.44 had a sensitivity of 60%–70%, specificity of 67%–74%, positive likelihood ratio (+LR) of 2.13–2.28, and negative likelihood ratio (-LR) of 0.44–0.54 for predicting TB normalization within 6 weeks after endobiliary stent placement (Table 4).

**Table 4:** The diagnostic performance of the risk score for predicting TB normalization within 6 weeks after endobiliary stent placement.

**The derivation cohort**						
**The risk score**	**Sensitivity (%) (95% CI)**	**Specificity (%) (95% CI)**	**PPV (%) (95% CI)**	**NPV (%) (95% CI)**	**+LR (95% CI)**	**-LR (95% CI)**
Low score of -4.18	90.1 (82.1–95.4)	45.4 (39.2–51.7)	36.6 (73.1–77.6)	92.9 (47.9–71.6)	1.65 (1.45–1.88)	0.22 (0.12–0.41)
Optimal score of -2.44	70.3 (59.8–79.5)	66.9 (60.8–72.6)	42.7 (37.4–48.1)	86.6 (82.3–89.9)	2.13 (1.71–2.65)	0.44 (0.32–0.62)
High score of 1.30	17.6 (10.4–27.0)	98.9 (96.7–99.8)	84.2 (61.4–94.7)	77.4 (75.7–79.0)	15.24 (4.54–51.1)	0.83 (0.76–0.92)
**The validation cohort**						
**The risk score**	**Sensitivity (%) (95% CI)**	**Specificity (%) (95% CI)**	**PPV (%) (95% CI)**	**NPV (%) (95% CI)**	**+LR (95% CI)**	**-LR (95% CI)**
Low score of -4.18	80.0 (59.3–93.2)	47.3 (36.7–58.0)	29.4 (24.0–35.4)	89.6 (79.2–95.1)	1.52 (1.15–2.00)	0.42 (0.19–0.95)
Optimal score of -2.44	60.0 (38.7–78.9)	73.6 (63.3–82.3)	38.5 (38.1–50.0)	87.0 (80.3–91.7)	2.28 (1.42–3.64)	0.54 (0.33–0.89)
High score of 1.30	20.0 (6.8–40.7)	98.9 (94.0–100.0)	83.3 (38.0–97.6)	81.8 (78.7–84.6)	18.20 (2.23–148.8)	0.81 (0.66–0.99)

+LR, positive likelihood ratio; -LR, negative likelihood ratio; NPV, negative predictive value; PPV, positive predictive value

We further explored different cutoffs of the TB normalization scoring system to discriminate the subgroups of patients with the lowest and highest chance of clinical response to endobiliary stent placement (Table 4). Patients in the derivation cohort were categorized into 3 groups using cutoffs of -4.18 and 1.30. Patients scoring ≥ 1.30 were placed in the high-likelihood group; 84% of these patients had a correct prediction. Patients with a cutoff of 1.30 had a high likelihood of TB normalization after endobiliary stent placement (PPVs of 83%–84%). Patients scoring < -4.18 were assigned to the low-likelihood group. The absence of TB normalization after endobiliary stenting could be ruled out with high certainty (NPVs of 90%–93%).

## Discussion

Our study assessed clinical success using different criteria at 2 points after endobiliary stenting: (1) a 50% reduction in TB levels within 2 weeks and (2) normalization of TB levels within 6 weeks. Most patients who achieved an early response had TB normalization. Nonetheless, 66.2% of cases with an early improvement in bilirubin levels failed to achieve normalization of bilirubin at 6 weeks. This finding signified that a TB regression exceeding 50% could not guarantee TB normalization. Additionally, 8.8% of cases without an early response at 2 weeks eventually achieved TB normalization within 6 weeks. Therefore, the significant variables at 6 weeks were the essential predictors that influenced the clinical outcomes after stenting.

The location of the biliary obstruction on cross-sectional imaging and the stent types were significantly associated with early clinical success and TB normalization after stenting. Extrahepatic biliary obstruction influenced clinical success, whereas hilar obstruction showed an inverse association. Because the drainage strategy for hilar obstruction is complex, patients with this condition were less likely to achieve clinical success after stenting than those with extrahepatic obstruction. Thus, pancreatic cancer was significantly associated with clinical success at 2 and 6 weeks. In contrast, hilar cholangiocarcinoma was associated with clinical failure within 2 weeks after endobiliary stent placement.

Regarding the stent types, we observed that SEMSs were used more frequently in patients with clinical success, whereas plastic stents were used more often in those without clinical success. Moreover, most patients who achieved early clinical success and TB normalization had a lower rate of stent dysfunction and longer stent patency time. This finding suggests that SEMSs were more effective than plastic stents for biliary decompression. Nevertheless, the number of stents did not influence clinical success at either 2 or 6 weeks after stenting.

In the current investigation, pre-endoscopic TB levels were a significant parameter associated with TB normalization within 6 weeks after endobiliary stenting. Weston et al. also reported that a pre-stenting bilirubin level of more than 10 mg/dL was significantly associated with a slow rate of bilirubin normalization and required more than 6 weeks for bilirubin levels to drop below 2 mg/dL [18]. Thus, the duration of 2 weeks after endoscopic drainage might not be the optimal time point to preclude patients without TB resolution from chemotherapy, especially for patients with high pre-endoscopic TB levels. Additionally, Weston and colleagues demonstrated that patients failed to achieve clinical success after endobiliary stent placement if they had certain conditions. They were evidence of advanced diseases, some degree of declined liver function (including diffuse or multiple liver metastases), an international normalized ratio above 1.4, and recent prior chemotherapy. In our study, hilar obstruction and a plastic stent were independent factors in the delayed normalization of TB following stenting. Therefore, the baseline TB level alone could not accurately predict the clinical response to endobiliary stenting in patients with unresectable MBO.

The pre-endoscopic ALP level and the presence of peritoneal carcinomatosis were also significant baseline factors associated with clinical success within 2 weeks after stenting. However, these predictors showed no significant association with the 6-week TB normalization. With the pre-endoscopic ALP, a prior study found that a less than 50% decrease in ALP levels 2 weeks after stenting was a predictor for metallic stent occlusion in unresectable pancreatic cancer patients [31]. However, our study did not emphasize the degree of serum ALP regression after stenting. Our results revealed that a high pre-endoscopic ALP level could predict clinical success within 2 weeks after stenting.

We hypothesized that patients with high-grade biliary obstruction, presenting as elevated initial serum bilirubin and ALP, would initially show a respectable clinical response but not at 6 weeks. In practice, the rate of biochemical normalization probably depends on multiple factors. They might include the duration of biliary tract obstruction prior to presentation, the nature of the diseases, liver function, the location of the biliary obstruction, and evidence of advanced disease. In our study, peritoneal carcinomatosis had an inverse association with 2-week clinical success but not with 6-week clinical success. The reason is that 15.4% of patients with advanced-stage cancer and peritoneal carcinomatosis died between 2 and 6 weeks after receiving endobiliary drainage.

This study offers simple risk scores for predicting the clinical success of TB resolution after endobiliary stent placement. The scores draw upon the parameters that reflected the degree of biliary tract obstruction. These are liver function before biliary decompression, the extent of obstruction, and the stent type planned by the endoscopist for biliary decompression. The “bilirubin improvement score” for predicting clinical success within 2 weeks and the “bilirubin normalization score” for predicting clinical success within 6 weeks were verified in the validation cohort and showed similar discriminative powers. The bilirubin improvement score for predicting clinical success at week 2 had a lower discriminative ability for excluding patients with clinical failure using a low cutoff than the risk score at week 6. Thus, the risk score for predicting TB normalization within 6 weeks after stenting is more impactful in clinical decision-making for managing unresectable MBO. To our knowledge, the prediction model for TB normalization after ERCP-guided endobiliary decompression in patients with unresectable MBO has not been reported in prior studies. However, Eaton et al. proposed a prediction model for jaundice resolution in patients with primary sclerosing cholangitis who underwent ERCP-guided biliary decompression. Similar to our study, extrahepatic obstruction was predictive of jaundice resolution. The model by Easton et al. provided an AUROC of 0.74 and 0.67 in the derivation and validation cohorts, respectively [32].

The cutoffs were based on assessing sensitivity, specificity, PPV, and NPV to provide clinical application according to the scenarios. For example, patients with a risk score > 1.3 would be highly likely to show a clinical response to endobiliary drainage. However, patients scoring < -4.18 would have a low likelihood of achieving TB normalization. These patients may not require ERCP for palliative care except for biliary tract infection management. Patients scoring -4.18 to 1.30 were placed in an intermediate-risk group. Their performance status and the physician’s clinical judgment should be considered for further management, and counseling with the patients and their relatives is essential. Close observation for adverse events or clinical deterioration is crucial, and prompt biliary decompression should be offered in cases of superimposed infection.

The strengths of this study are its large patient cohort of unresectable MBO patients undergoing endobiliary stenting with complete follow-up and clinical data. We identified the predictors and developed a risk score for 2 time points after endobiliary stenting, an innovative approach, to determine the best scoring system for predicting clinical success. The score consisted of the predictors obtained from imaging findings (extrahepatic bile duct obstruction), routine laboratory tests, and the type of endobiliary stent. Upon validation with a different set of patients, the risk score showed good prediction with acceptable diagnostic performance in patients from both derivation and validation cohorts. A weblink for the score calculator is provided for real-time use of the scoring system in clinical practice.

Nonetheless, some limitations of this study should be noted. Patients with unresectable MBO due to hepatocellular carcinoma and patients with advanced cirrhosis were excluded. Thus, the findings do not apply to patients with underlying advanced liver diseases. Moreover, external validation is required to determine the reliability of the developed clinical risk score.

## Conclusions

Establishing an optimal palliative drainage strategy using a simple pre-endoscopic scoring scheme comprised of liver biochemistry, site of bile duct obstruction, and the type of endobiliary stent to be used may be beneficial in identifying patients with unresectable MBO suited for palliative stenting.

## Supporting information

S1 Table Comparison of baseline characteristics of patients with and without 50% total bilirubin reduction within 2 weeks after endoscopic drainage in the derivation cohort. (DOCX)

S2 Table Cholangiographic findings and endoscopic interventions of patients with and without 50% total bilirubin reduction within 2 weeks after stenting in the derivation cohort. (DOCX)

S3 Table Diagnostic performance of the risk score for predicting clinical success within 2 weeks after endobiliary stent placement. (DOCX)

S4 Table Comparison of baseline characteristics of patients with and without bilirubin normalization within 6 weeks after endoscopic drainage in the derivation cohort. (DOCX)

S5 Table Cholangiographic findings and endoscopic interventions of patients with and without bilirubin normalization within 6 weeks after endoscopic drainage in the derivation cohort. (DOCX)

## Abbreviations

ALP: alkaline phosphatase
AUROC: area under the receiver operating characteristic curve
ERCP: endoscopic retrograde cholangiopancreatography
INR: international normalized ratio
LR−: negative likelihood ratio
LR+: positive likelihood ratio
MBO: malignant biliary obstruction
NPV: negative predictive value
PPV: positive predictive value
PTBD: percutaneous transhepatic biliary drainage
SEMS: self-expandable metallic stent
TB: total bilirubin
ULN: upper limit of normal
