# Supplementary material for: Development and validation of a risk score for predicting clinical success after endobiliary stenting for malignant biliary obstruction
Source: PLoS One. 2022 Aug 19;17(8):e0272918. doi: 10.1371/journal.pone.0272918 (PMC9390920; doi:10.1371/journal.pone.0272918)
Supplement: S1 Table — (DOCX) [file pone.0272918.s001.docx]

**Table S1.** Comparison of baseline characteristics of patients with and without 50% total bilirubin reduction within 2 weeks after endoscopic drainage in the derivation cohort

| **Characteristics** | **50% TB reduction**  **(N = 270)** | **No 50% TB reduction**  **(N = 113)** | ***P* value** |
| --- | --- | --- | --- |
| Male gender, n (%) | 132 (48.9%) | 64 (56.6%) | 0.167 |
| Age, years | 64.0 ± 13.0 | 62.0 ± 13.2 | 0.222 |
| **Type of malignancy** |  |  |  |
| Cholangiocarcinoma, n (%) | 118 (43.7%) | 67 (59.3%) | **0.005** |
| Intrahepatic cholangiocarcinoma | 21 (17.8%) | 10 (15.0%) | 0.338 |
| Hilar cholangiocarcinoma | 54 (54.2%) | 44 (65.7%) | **< 0.001** |
| Extrahepatic cholangiocarcinoma | 33 (28.0%) | 13 (19.4%) | 0.965 |
| Pancreatic cancer | 95 (35.2%) | 23 (20.4%) | **0.001** |
| Gallbladder cancer | 23 (8.5%) | 9 (8%) | 0.521 |
| Ampullary cancer | 15 (5.6%) | 0 (0) | **0.004** |
| **Clinical presentation, n (%)** |  |  |  |
| Abdominal pain | 146 (54.1%) | 68 (60.2%) | 0.273 |
| Jaundice | 240 (88.9%) | 101 (89.4%) | 0.888 |
| Fever | 16 (5.9%) | 7 (6.2%) | 0.920 |
| Ascending cholangitis | 54 (20.0%) | 21 (18.6%) | 0.750 |
| **Pre-endoscopic laboratory** |  |  |  |
| Hemoglobin, g/dL | 10.7 ± 3.2 | 10.4 ± 2.0 | 0.361 |
| Platelet x10^3^/microliter | 320 (258–405) | 319 (258–397) | 0.945 |
| INR | 1.4 ± 0.5 | 1.4 ± 0.6 | 0.481 |
| Total bilirubin, mg/dL | 18.3 (12.7–26.8) | 17.2 (10.5–23.8) | **0.046** |
| Albumin, g/dL | 3.3 ± 0.6 | 3.1 ± 0.7 | 0.056 |
| Alkaline phosphatase, IU/L | 523 (301–727) | 408 (281–577) | **0.030** |
| Creatinine, mg/dL | 0.8 (0.6–0.9) | 0.8 (0.6–1.0) | 0.138 |
| **Cross-sectional imaging** |  |  |  |
| Size of obstructive tumor, cm | 3.5 (2.5–5.0) | 4.0 (2.5–6.5) | 0.153 |
| Hilar obstruction | 84 (31.1%) | 59 (52.2%) | **< 0.001** |
| Non-hilar obstruction | 186 (68.9%) | 54 (47.8%) | **< 0.001** |
| Intrahepatic obstruction | 8 (3.0%) | 7 (6.2%) | 0.153 |
| Extrahepatic obstruction | 177 (65.6%) | 47 (41.6%) | **< 0.001** |
| Combined obstruction | 6 (2.2%) | 9 (8%) | **0.017** |
| Portal vein invasion, n (%) | 71 (26.3%) | 41 (36.3%) | **0.050** |
| Distant metastasis, n (%) | 153 (56.7%) | 67 (59.3%) | 0.636 |
| Liver metastasis, n (%) | 86 (31.9%) | 44 (38.9%) | 0.182 |
| Peritoneal carcinomatosis, n (%) | 28 (10.4%) | 21 (18.6%) | **0.028** |
| **Post-stenting outcome** |  |  |  |
| Chemotherapy after stenting, n (%) | 52 (19.3%) | 13 (11.5%) | 0.065 |

INR, international normalized ratio; TB, total bilirubin

Data are presented as mean ± standard deviation, median (interquartile range), or number (proportion) of patients with a condition.
