# Supplementary material for: Development and validation of a risk score for predicting clinical success after endobiliary stenting for malignant biliary obstruction
Source: PLoS One. 2022 Aug 19;17(8):e0272918. doi: 10.1371/journal.pone.0272918 (PMC9390920; doi:10.1371/journal.pone.0272918)
Supplement: S2 Table — (DOCX) [file pone.0272918.s002.docx]

**Table S2.** Cholangiographic findings and endoscopic interventions of patients with and

without 50% total bilirubin reduction within 2 weeks after stenting in the derivation cohort

| **Characteristics** | **50% TB reduction**  **(N = 270)** | **No 50% TB reduction**  **(N = 113)** | ***P* value** |
| --- | --- | --- | --- |
| Length of biliary stricture, mm | 20 (13.0–30.0) | 16 (14.0–28.0) | 0.178 |
| Diameter of intrahepatic biliary dilatation, mm | 15.4 ± 6.2 | 13.7 ± 4.7 | 0.066 |
| Diameter of extrahepatic biliary dilatation, mm | 17.6 ± 7.0 | 17.0 ± 7.1 | 0.620 |
| Presence of either plastic or metallic stent | 83 (30.7%)/187 (69.3%) | 55 (48.7%)/58 (51.3%) | **0.021** |
| One-stent placement | 257 (95.2%) | 105 (92.9%) | 0.375 |
| Plastic stent placement, n (%) | 79 (29.3%) | 52 (46.0%) | **0.001** |
| Metallic stent placement, n (%) | 178 (65.9%) | 53 (46.9%) | **0.001** |
| - Uncovered SEMS | 167 (61.9%) | 52 (46.0%) | **0.006** |
| - Fully covered SEMS | 8 (3.0%) | 0 (0) | 0.111 |
| - Partial covered SEMS | 3 (1.1%) | 1 (0.9%) | 1.000 |
| Two-stent placements, n (%) | 13 (4.8%) | 8 (7.1%) | 0.375 |
| Two metallic stents | 8 (3%) | 5 (4.4%) | 1.000 |
| Two plastic stents | 4 (1.5%) | 3 (2.7%) | 1.000 |
| One metallic and one plastic stent | 1 (0.4%) | 0 (0) | 1.000 |
| Stent dysfunction, n (%) | 68 (25.3%) | 34 (30.1%) | 0.322 |
| Stent patency time, days | 83.0 (56.0–194.0) | 28.5 (17.0–51.0) | **< 0.001** |

ERCP, endoscopic retrograde cholangiopancreatography; SEMS, self-expandable metallic stent

Data are presented as mean ± standard deviation, median (interquartile range), or number (proportion) of patients with a condition.
