# Supplementary material for: Development and validation of a risk score for predicting clinical success after endobiliary stenting for malignant biliary obstruction
Source: PLoS One. 2022 Aug 19;17(8):e0272918. doi: 10.1371/journal.pone.0272918 (PMC9390920; doi:10.1371/journal.pone.0272918)
Supplement: S3 Table — (DOCX) [file pone.0272918.s003.docx]

**Table S3.** Diagnostic performance of the risk score for predicting clinical success within 2 weeks after endobiliary stent placement.

| **The derivation cohort** | | | | | | |
| --- | --- | --- | --- | --- | --- | --- |
| **The risk score** | **Sensitivity (%)**  **(95% CI)** | **Specificity (%)**  **(95% CI)** | **PPV (%)**  **(95% CI)** | **NPV (%)**  **(95% CI)** | **+LR**  **(95% CI)** | **-LR**  **(95% CI)** |
| Low score of 0.71 | 92.2  (88.4–94.9) | 29.2  (21.6–38.2) | 75.7  (70.8–80.0) | 61.1  (47.8–73.0) | 1.30  (1.15–1.47) | 0.27  (0.16–0.44) |
| Optimal score of 4.07 | 70.0 (64.3–84.1) | 56.6  (47.4–65.4) | 79.4  (73.8–84.1) | 44.1  (36.3–52.3) | 1.61  (1.29–2.02) | 0.53  (0.42–0.68) |
| High score of 8.98 | 23.7  (19.0–29.1) | 90.3  (83.4–94.5) | 85.3  (75.6–91.6) | 33.1  (28.1–38.6) | 2.44  (1.34–4.44) | 0.85  (0.77–0.93) |
| **The validation cohort** | | | | | | |
| **The risk score** | **Sensitivity (%)**  **(95% CI)** | **Specificity (%)**  **(95% CI)** | **PPV (%)**  **(95% CI)** | **NPV (%)**  **(95% CI)** | **+LR**  **(95% CI)** | **-LR**  **(95% CI)** |
| Low score of 0.71 | 91.6  (84.3–95.7) | 18.2  (8.6–34.4) | 76.3  (67.7–83.2) | 42.9  (21.4–67.4) | 1.12 (0.94–1.33) | 0.46  (0.17–1.24) |
| Optimal score of 4.07 | 67.4  (57.4–76.0) | 51.5  (35.2–67.5) | 80.0  (70.0–87.3) | 35.4  (23.4–49.6) | 1.39  (0.96–2.03) | 0.63  (0.41–0.98) |
| High score of 8.98 | 24.2  (16.7–33.7) | 90.9  (76.4–96.9) | 88.5  (71.0–96.0) | 29.4  (21.4–38.9) | 2.66  (0.86–8.28) | 0.83  (0.71–0.98) |

-LR, negative likelihood ratio; +LR, positive likelihood ratio; NPV, negative predictive value; PPV, positive predictive value
