# Supplementary material for: Development and validation of a risk score for predicting clinical success after endobiliary stenting for malignant biliary obstruction
Source: PLoS One. 2022 Aug 19;17(8):e0272918. doi: 10.1371/journal.pone.0272918 (PMC9390920; doi:10.1371/journal.pone.0272918)
Supplement: S4 Table — (DOCX) [file pone.0272918.s004.docx]

**Table S4.** Comparison of baseline characteristics of patients with and without bilirubin normalization within 6 weeks after endoscopic drainage in the derivation cohort.

| **Characteristics** | **Bilirubin normalization^a^**  **(N = 91)** | **No bilirubin normalization**  **(N = 201)** | ***P* value** |
| --- | --- | --- | --- |
| Male gender, n (%) | 47 (51.6%) | 98 (48.8%) | 0.647 |
| Age, years | 63.7 ± 13.1 | 63.1 ± 13.4 | 0.678 |
| **Type of malignancy** |  |  |  |
| Cholangiocarcinoma, n (%) | 36 (39.6%) | 102 (50.7%) | 0.076 |
| Intrahepatic cholangiocarcinoma | 4 (4.4%) | 17 (8.5%) | 0.213 |
| Hilar cholangiocarcinoma | 23 (25.3%) | 74 (36.8%) | 0.052 |
| Extrahepatic cholangiocarcinoma | 11 (12.1%) | 19 (9.5%) | 0.492 |
| Pancreatic cancer | 37 (40.7%) | 56 (27.9%) | **0.030** |
| Gallbladder cancer | 5 (5.5%) | 16 (8.0%) | 0.450 |
| Ampullary cancer | 4 (4.4%) | 11 (5.5%) | 0.699 |
| **Clinical presentation, n (%)** |  |  |  |
| Abdominal pain | 46 (50.5%) | 113 (56.2%) | 0.368 |
| Jaundice | 77 (84.6%) | 186 (92.5%) | **0.036** |
| Fever | 10 (11.0%) | 10 (5.0%) | 0.060 |
| Ascending cholangitis | 17 (18.7%) | 39 (19.4%) | 0.885 |
| **Pre-endoscopic laboratory** |  |  |  |
| Hemoglobin, g/dL | 10.6 ± 1.8 | 10.8 ± 3.6 | 0.845 |
| Platelet x 10^3^/microliter | 319 (254–403) | 327 (296–396) | 0.560 |
| INR | 1.2 ± 0.3 | 1.4 ± 0.5 | **0.011** |
| Total bilirubin, mg/dL | 12.8 ± 7.6 | 21.1 ± 9.07 | **< 0.001** |
| Albumin, g/dL | 3.4 ± 0.6 | 3.2 ± 0.6 | **0.008** |
| Alkaline phosphatase, IU/L | 463 (305–732) | 446 (276–660) | 0.452 |
| Creatinine, mg/dL | 0.8 (0.7–0.9) | 0.8 (0.6–1.0) | 0.840 |
| **Cross-sectional imaging** |  |  |  |
| Size of obstructive tumor, cm | 3.3 (2.4–4.5) | 3.6 (2.4–5.5) | 0.091 |
| Hilar obstruction | 28 (30.8%) | 88 (43.8%) | **0.035** |
| Non-hilar obstruction | 63 (69.2%) | 113 (56.2%) | **0.035** |
| Intrahepatic obstruction | 1 (1.1%) | 4 (2.0%) | 1.000 |
| Extrahepatic obstruction | 62 (68.1%) | 109 (54.2%) | **0.026** |
| Combined obstruction | 3 (3.3%) | 11 (5.5%) | 0.560 |
| Portal vein invasion, n (%) | 23 (25.3%) | 61 (30.3%) | 0.375 |
| Distant metastasis, n (%) | 50 (54.9%) | 108 (53.7%) | 0.847 |
| Liver metastasis, n (%) | 32 (35.2%) | 58 (28.9%) | 0.280 |
| Peritoneal carcinomatosis, n (%) | 9 (9.9%) | 24 (11.9%) | 0.608 |
| **Post-stenting outcomes** |  |  |  |
| 50% TB reduction in 2 weeks, n (%) | 83 (91.2%) | 133 (66.2%) | **< 0.001** |
| Chemotherapy after stenting, n (%) | 36 (39.6%) | 25 (12.4%) | **< 0.001** |

INR, international normalized ratio; TB, total bilirubin

Data are presented as mean ± standard deviation, median (interquartile range), or number (proportion) of patients with a condition.

^a^ Defined by a normalization of TB levels of less than 1.2 mg/dL within six weeks after ERCP-guided endobiliary stent placement
