# Supplementary material for: Development and validation of a risk score for predicting clinical success after endobiliary stenting for malignant biliary obstruction
Source: PLoS One. 2022 Aug 19;17(8):e0272918. doi: 10.1371/journal.pone.0272918 (PMC9390920; doi:10.1371/journal.pone.0272918)
Supplement: S5 Table — (DOCX) [file pone.0272918.s005.docx]

**Table S5.** Cholangiographic findings and endoscopic interventions of patients with and

without bilirubin normalization within 6 weeks after endoscopic drainage in the derivation

cohort.

| **Characteristics** | **Bilirubin normalization^a^**  **(N = 91)** | **No Bilirubin normalization**  **(N = 201)** | ***P* value** |
| --- | --- | --- | --- |
| Length of biliary stricture, mm | 20 (13.0–25.0) | 20 (13.0–30.0) | 0.933 |
| Diameter of intrahepatic biliary dilatation, mm | 16.3 ± 7.2 | 14.4 ± 4.8 | 0.181 |
| Diameter of extrahepatic biliary dilatation, mm | 17.3 ± 7.6 | 18.0 ± 6.9 | 0.131 |
| Presence of either plastic or metallic stent | 26 (28.6%)/65 (71.4%) | 86 (42.8%)/115 (57.2%) | **0.021** |
| One-stent placement | 85 (93.4%) | 190 (94.5%) | 0.705 |
| Plastic stent placement, n (%) | 24 (26.4%) | 81 (40.3%) | **0.022** |
| Metallic stent placement, n (%) | 61 (67.0%) | 109 (54.2%) | **0.040** |
| - Uncovered SEMS | 55 (60.4%) | 103 (51.2%) | 0.144 |
| - Fully covered SEMS | 5 (5.5%) | 3 (1.5%) | 0.052 |
| - Partial covered SEMS | 1 (1.1%) | 3 (1.5%) | 1.000 |
| Two-stent placements, n (%) | 6 (6.6%) | 11 (5.5%) | 0.705 |
| Two metallic stents | 4 (4.4%) | 5 (2.5%) | 0.467 |
| Two plastic stents | 2 (2.2%) | 5 (2.5%) | 1.000 |
| One metallic and one plastic stent | 0 (0) | 1 (9.1%) | 1.000 |
| Stent dysfunction, n (%) | 25 (27.5%) | 67 (33.3%) | 0.318 |
| Stent patency time, days | 119 (73.0–197.0) | 60.5 (28.5–89.0) | **0.003** |

ERCP, endoscopic retrograde cholangiopancreatography; SEMS, self-expandable metallic stent

Data are presented as mean ± standard deviation, median (interquartile range), or number (proportion) of patients with a condition.

^a^ Defined by a normalization of TB level of less than 1.2 mg/dL within six weeks after ERCP-guided endobiliary stent placement
